# Thermoplasmonic Controlled Optical Absorber Based on a Liquid Crystal Metasurface

**DOI:** 10.1021/acsami.3c09896

**Published:** 2023-10-10

**Authors:** Francesca Petronella, Tristan Madeleine, Vincenzo De Mei, Federica Zaccagnini, Marinella Striccoli, Giampaolo D’Alessandro, Mariacristina Rumi, Jonathan Slagle, Malgosia Kaczmarek, Luciano De Sio

**Affiliations:** † National Research Council of Italy, Institute of Crystallography, CNR-IC, Rome Division, Area della Ricerca Roma 1 Strada Provinciale 35d, n. 9, 00010 Montelibretti (RM), Italy; ‡ School of Mathematical Science, University of Southampton, Southampton SO17 1BJ, United Kingdom; § Department of Medico-Surgical Sciences and Biotechnologies Sapienza, University of Rome, 00185 Latina, Italy; ∥ National Research Council of Italy, Institute of Chemical and Physical Processes CNR-IPCF Bari Division, Via Orabona 4, 70126 Bari, Italy; ⊥ Materials and Manufacturing Directorate, Air Force Research Laboratory, Wright-Patterson Air Force Base, Ohio 45433-7707, United States; # National Research Council of Italy, Licryl, Institute NANOTEC, 87036 Arcavacata di Rende, Italy; ▽ School of Physics and Astronomy, University of Southampton, Southampton SO17 1BJ, United Kingdom

**Keywords:** metasurface, liquid crystals, active control, thermoplasmonics, lithography-free, colloidal nanoparticles

## Abstract

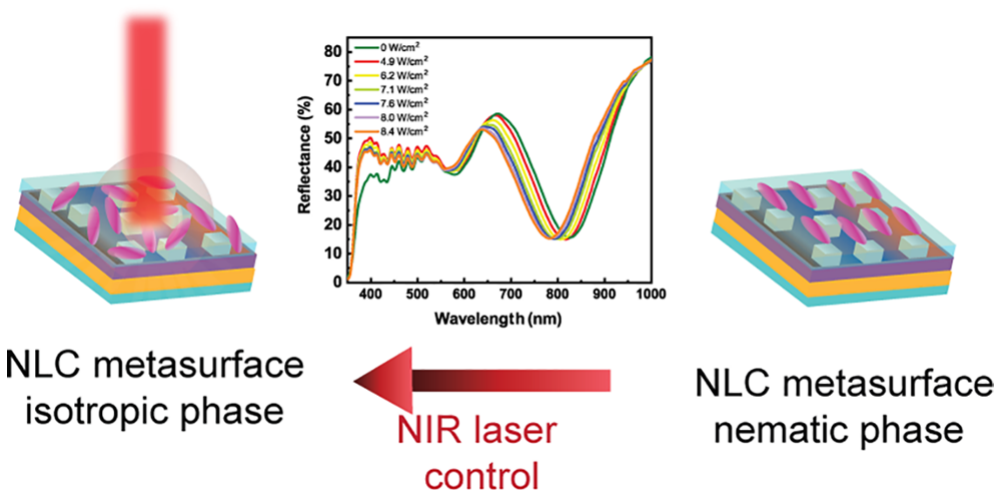

Metasurfaces can be realized by organizing subwavelength elements (e.g., plasmonic nanoparticles) on a reflective surface covered with a dielectric layer. Such an array of resonators, acting collectively, can completely absorb the resulting resonant wavelength. Unfortunately, despite the excellent optical properties of metasurfaces, they lack the tunability to perform as adaptive optical components. To boost the utilization of metasurfaces and realize a new generation of dynamically controlled optical components, we report our recent finding based on the powerful combination of an innovative metasurface-optical absorber and nematic liquid crystals (NLCs). The metasurface consists of self-assembled silver nanocubes (AgNCs) immobilized on a 50 nm thick gold layer by using a polyelectrolyte multilayer as a dielectric spacer. The resulting optical absorbers show a well-defined reflection band centered in the near-infrared of the electromagnetic spectrum (750–770 nm), a very high absorption efficiency (∼60%) at the resonant wavelength, and an elevated photothermal efficiency estimated from the time constant value (34 s). Such a metasurface-based optical absorber, combined with an NLC layer, planarly aligned via a photoaligned top cover glass substrate, shows homogeneous NLC alignment and an absorption band photothermally tunable over approximately 46 nm. Detailed thermographic studies and spectroscopic investigations highlight the extraordinary capability of the active metasurface to be used as a light-controllable optical absorber.

## Introduction

### Metasurface Properties and Preparation

The field of metamaterials, artificial materials that exhibit unique optical properties not otherwise found in nature,^1^ has unlocked unprecedented opportunities for manipulating the light’s amplitude, phase, and polarization at the nanoscale.

Among metamaterials, groundbreaking technological opportunities arise from metasurfaces, a class of planar optical devices with novel peculiar effects.^2−4^ Metasurfaces typically consist of a thin dielectric spacer sandwiched between a metal film and an array of metal subwavelength elements (such as metallic nanostructures).^5^

The plasmonic resonances of metallic nanostructures are particularly interesting for metasurface fabrication, originating from a strong subwavelength light–matter interaction. The localized surface plasmon resonance (LSPR) of individual metal nanoparticles (NPs) is associated with an enhanced local electric field, absorption, and light scattering at the resonance wavelength.^1,6,7^ However, owing to the significant radiative and Ohmic damping, an LSPR effect is usually characterized by low-quality factors, such as wide spectral line widths. LSPR quality factors can be improved by optically coupling the LSPRs of an individual metal NP with those of other NPs, all suitably organized in arrays if the array spacing is comparable to the incident radiation wavelength. The result is the appearance of new hybridized photonic-plasmonic modes, known as surface lattice resonances (SLRs), which exhibit narrow spectral line widths, along with relatively high-quality factors, giving rise to a metasurface.^8−10^

However, the most common metasurface fabrication protocols use top-down approaches such as sputtering and electron beam lithography.^3,4,11,12^ The use of top-down lithography techniques inherently limits the scalability of metasurfaces to large areas, hindering the possibility of achieving low-cost, large-scale devices or conformal metasurfaces. To fill this gap, Moreau and co-workers^13^ and then Akselrod and co-workers^14^ have reported on the possibility of employing colloidal plasmonic NPs as building blocks to realize plasmonic metasurfaces by exploiting self-assembly techniques, thus overcoming the limits of top-down physical technologies.^14−16^

### Active Metasurfaces

To fully exploit the application potential of metasurfaces, the dynamic control of their optical properties is needed to achieve a reconfigurable meta-device.^7^ Integrating a nematic liquid crystal (NLC) active layer is an attractive approach to realize a dynamically tunable metasurface. NLCs are anisotropic materials that show large optical birefringence and have been widely used to manipulate light propagation through external stimuli (e.g., electric/magnetic fields, temperature variations, optical fields, etc.). Recent studies have reported on realizing in NLC-based metasurfaces fast-switching photonic devices at optical communication wavelengths, beam-steerable antennas, tunable metalenses, polarization control elements, high-resolution displays, and sensing applications.^3,4,11,12,17−20^ For instance, Sharma and co-workers realized an electrically switchable plasmonic metasurface activated by an NLC layer. The authors observed electrically tunable SLR modes in the near-infrared (NIR) range.^11^ The same research group achieved an electrically switchable plasmonic metasurface with an NLC by integrating plasmonic metasurface based color tags.^11^ In a different approach, Liu and co-workers^12^ have developed an electrically tunable metasurface for polarization conversion at visible frequencies. Wang et al. have functionalized gold metasurfaces with an NLC film for display and modulator applications, with high contrast, large modulation depth, and well-evident switching effect in both the visible and NIR ranges.^21^

In the present work, we aim to realize a thermoplasmonic controlled active metasurface by a colloidal self-assembly approach, thus avoiding expensive nanofabrication techniques and using an NLC layer as an active material.

Inspired by the work of Akselrod and co-workers,^14^ and with some modification to the fabrication procedure, we report on self-assembled and light-controllable metasurfaces that exploit the stimuli responsiveness of a thermotropic NLC film. In particular, for fabricating the metasurface, a polyelectrolyte multilayer was used as a dielectric spacer to host an array of silver nanocubes (AgNCs) on a 50 nm thick gold layer; then, an NLC film was integrated to introduce thermoplasmonic tunability. The NLC optical properties are controlled by the thermoplasmonic heating produced by irradiating the metasurface with a resonant light source (NIR radiation) that matches the metasurface absorbance band. Consequently, the metasurface absorption band’s wavelength can be modulated on demand and remotely by simply varying the power density of the NIR laser impinging the NLC metasurface. The proposed approach merges the benefit of colloidal self-assembly techniques with the tunability of an NLC active layer, thus opening a new avenue for fabricating low-cost, conformal, and scalable active devices.

## Experimental Section

### Materials

Gold-coated (Au) glass slides, 50 nm thick (1 cm × 1 cm), were purchased from AMSBIO. AgNCs (100 nm side, 1 mg) were purchased from Nanocomposix. The polyelectrolytes (PEs) poly(sodium 4-styrenesulfonate) (PSS, *M*_w_ 70000 Da), poly(allylamine hydrochloride) (PAH, *M*_w_ 50000 Da), and NLC E7 were purchased from Merck. Sodium chloride (NaCl) was purchased from Sigma-Aldrich. NOA 61 was purchased from Norlands, and the photoalignment azo dye (PAAD-72) was provided by Beam Engineering for Advanced Measurements Company. Millipore water was used in all of the procedures.

### Metasurface Preparation

The metasurface preparation followed the protocol reported in ref (14), with some modifications. First, Au glass slides were carefully washed with water and dried under a nitrogen flow. After that, the slides were alternatively immersed for 5 min in the positively charged PE (PAH, 1.6 mg/mL dissolved in 0.5 M NaCl) and in the negatively charged PE (PSS, 1.6 mg/mL, dissolved in 0.5 M NaCl), resulting in a PE multilayer (PEM) with the sequence PAH/PSS/PAH/PSS/PAH. An intermediate washing procedure performed by immersing the samples for 1 min in 0.5 M NaCl was carried out between two consecutive PE layer depositions. Subsequently, the PEM-functionalized Au substrates were rinsed with Millipore water, and then 5.6 μL of 0.9 mg/mL AgNCs, capped with polyvinylpyrrolidone, dispersed in water, was cast on the PEM. The Au substrates were then covered with a glass slide and stored at 4 °C for 24 h. After 24 h of incubation, the top cover substrates were removed and the samples were rinsed with Millipore water and gently dried under a nitrogen flow.

### Sensitivity to Refractive Index Changes

A glass cell was fabricated to investigate the metasurface sensitivity to refractive index (*n*) change by placing a few drops of the NOA 61 glue with 10 μm glass microbeads on the metasurface corners. The metasurface was then covered by a clean glass slide (1.5 × 1.5 cm) and finally sealed by low-power UV light irradiation for 1 min. The resulting uniform 10 μm thick gap was filled with NOA 61 as a representative high-*n* medium and ultimately used for optical and photothermal characterization.

### Numerical Simulation

The numerical simulations were performed using the electromagnetic waves and frequency domain interface of the optics module of the software COMSOL 5.6, solved with a direct solver.^22^ We considered 100 nm long AgNCs with rounded corners (curvature of 10 nm) deposited on a 10 nm PEM (*n* = 1.5418) on an infinitely extended Au substrate. The bottom and top parts of the integrating domain were perfectly matched layers, preventing any back reflections. The lateral sides of the domains were encoded with Floquet periodic boundary conditions. Without loss of generality, we chose an exciting light at normal incidence polarized in the *x* direction. The reflectance was extracted by using periodic port conditions. The lateral size of the domain, equivalent to the periodicity of the AgNC array, was 300 nm. Different values for periodicity can modify the optical response of the overall system. However, this choice of periodicity still allowed for an optical response dominated by isolated AgNCs.^23^ The optical properties of the background material were chosen to be those of air (*n* = 1), NOA 61 (*n* = 1.56), or E7 NLC (*n_o_* = 1.52, *n_e_* = 1.73).^24^ The NLC was assumed to be planarly aligned parallel to one of the AgNC axes. We computed the optical response upon illumination with unpolarized light by averaging the reflectance simulated with light polarized parallel and perpendicular to the orientation of the NLC molecules.

### Integration of a Nematic Liquid Crystal Layer

To fabricate the active metasurface NLC cell, PAAD-72 was spin-coated onto a clean glass substrate at 3000 rpm for 30 s, followed by baking in an oven at 100 °C for 10 min to complete solvent evaporation. Next, the glue NOA 61 with 10 μm glass microbeads was deposited on the corners of the metasurface. After that, the photoalignable top substrate was placed on the metasurface, and the cell was sealed by irradiation with UV light for 1 min. Finally, the resulting cell was illuminated with a polarized UV lamp for 10 min (3.5 mW/cm^2^) to induce a planar alignment direction perpendicular to the light polarization.^25^

### UV–Visible Reflectance Spectroscopy

Reflectance spectroscopy measurements were performed using a customized optical setup made of a USB spectrophotometer (USB 2000+XR1, Ocean Optics, FL, USA) equipped with an optical fiber suitable for reflectance measurement and with a UV–vis light source (CLS 100; Leica, Vienna, Austria). The samples were placed on a holder and controlled with *x*-*y*-*z* translation stages for alignment purposes. The customized optical setup (Figure 2d) was also equipped with a CW diode laser (Coherent Powerline) operating in the NIR range (λ = 808 nm) to determine the spectral response of the sample under laser illumination. The rectangular profile of the NIR laser beam was converted into an almost circular spot by utilizing a 20 cm focal length elliptical lens. The setup geometry was purposely realized for probing the sample area using the reflective fiber-coupled spectrophotometer that exactly overlapped the photoactivated area of the sample.

### Scanning Electron Microscopy

Scanning electron microscopy (SEM) analysis was performed with a Helios NanoLab 600 SEM instrument with an accelerating voltage of 5 kV and a working distance of 4.0 mm.

### Photothermal Characterization

The thermo-optical setup (Figure 4a) employed for evaluating the photothermal properties of the metasurface used a CW diode laser (Coherent Powerline) operating in the NIR range (λ = 808 nm). An elliptical lens with a 20 cm focal length was employed to convert the rectangular profile of the NIR laser into an almost circular spot. The spatial heating distribution and temperature profile were analyzed by a high-resolution thermal camera (FLIR, A655sc), generating thermal images of 640 × 480 pixels with an accuracy of ±0.2 °C. This worked seamlessly with proprietary software (FLIR ResearchIR Max) to record and process the thermal data acquired by the camera.

### Optical Microscopy and Polarized Optical Microscopy Characterization

Polarized optical microscopy (POM) analyses were performed using a reflective ZEISS Axiolab 5 microscope equipped with a high-resolution camera and a 50× magnification objective.

### Mueller Matrix Spectroscopic Polarimeter Characterization

The molecular axis orientation of the NLC film was characterized by a Mueller Matrix Spectroscopic Polarimeter (Axometrics AxoScan) equipped with a tunable visible source. The measurements were performed at 542 nm using a 100× magnification objective.

## Results and Discussion

### Metasurface Preparation and Characterization

**Figure 1:**
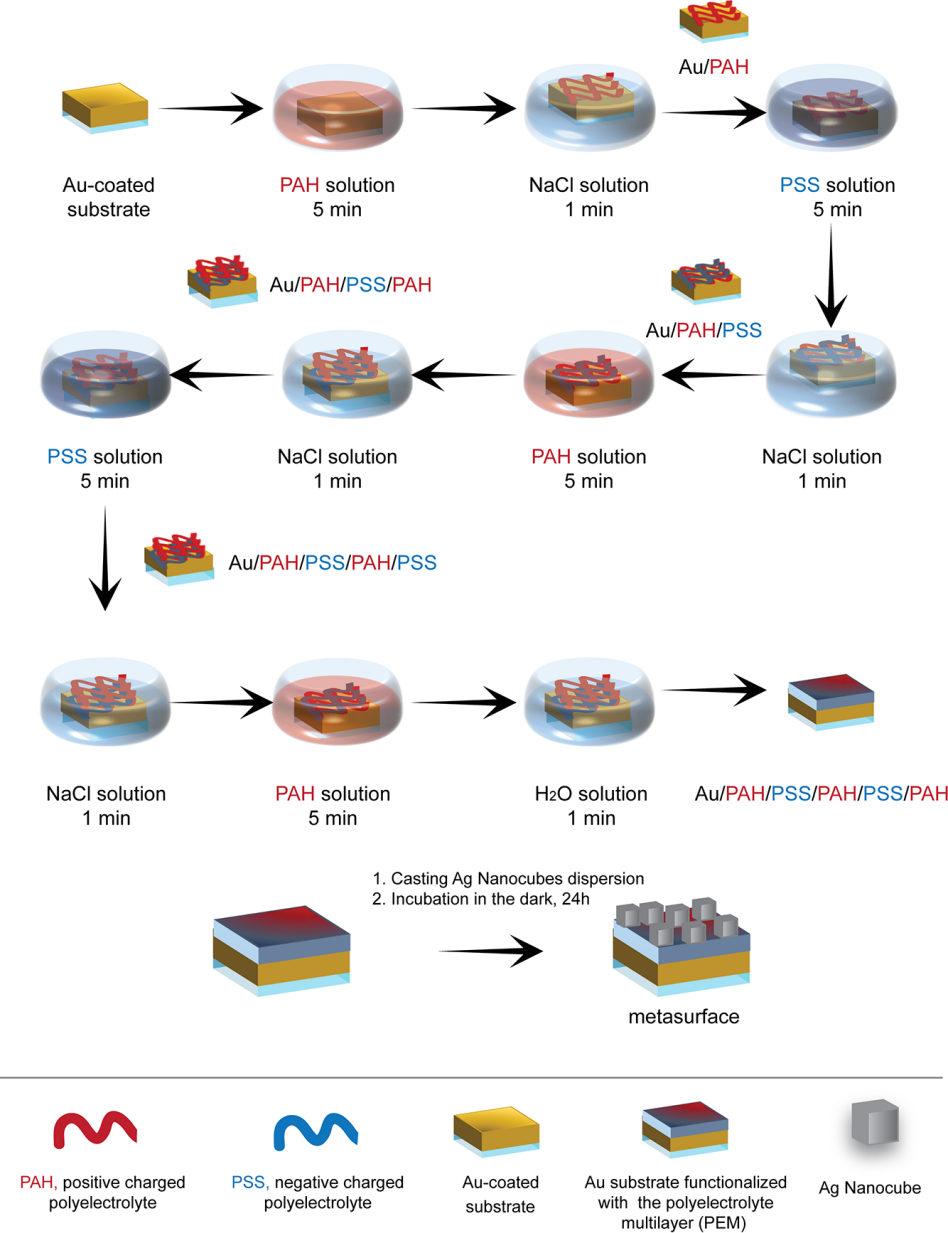
Schematic of the procedure adopted for preparing a colloidal metasurface by a bottom-up approach. The protocol involves functionalizing a 50 nm thick Au substrate by electrostatic layer by layer (eLbL) assembly with the PEM sequence PAH/PSS/PAH/PSS/PAH as a dielectric layer. Finally, after the final rinsing step with Millipore water, AgNCs were incorporated by drop-casting, and the system was incubated for 24 h at 4 °C in the dark and rinsed again with Millipore water before characterizations.

The procedure for the metasurface fabrication is based on the electrostatic layer-by-layer (eLbL) assembly technique, inspired by the work reported in ref (14). It essentially consists of depositing, in an alternating fashion, a positively charged PE (PAH) followed by a layer of a negatively charged PE (PSS) to build the PE multilayer (PEM) having the desired PE sequence, as reported in Figure 1. In the investigated experimental condition, each PE layer’s thickness lies between 5 and 10 nm.^26^

In the present work, the PEM consists of the sequence PAH/PSS/PAH/PSS/PAH, resulting in a dielectric spacer with nanometer precision over a large area (1 cm^2^) fabricated by a simple immersion-based procedure. The incorporation of AgNCs was realized by drop-casting the AgNC dispersion.

**Figure 2:**
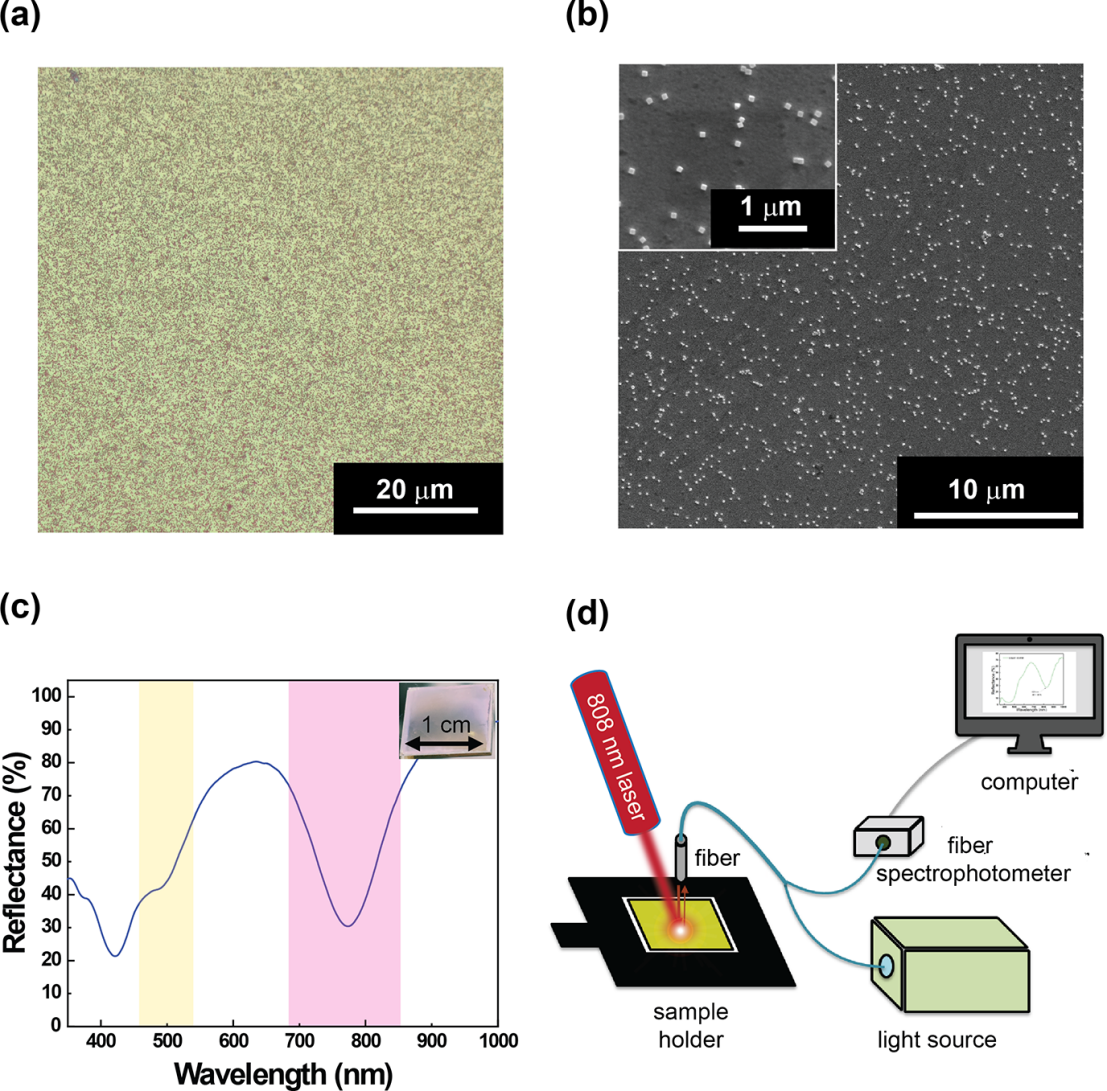
Metasurface morphology and optical characterization. Morphological analyses were performed by optical microscopy (a) and SEM microscopy (b). The inset of (b) shows the AgNCs monodispersed on the dielectric layer. Reflectance spectroscopy characterization of the metasurface. The inset reports a photograph of the metasurface (c). Schematic of the customized reflective fiber-coupled spectrophotometer equipped with an NIR laser used for the optical and thermospectrophotometric characterizations (d).

The reflective optical microscopy image of the as-prepared metasurface sample, reported in Figure 2a, showed the presence of AgNCs, evenly distributed over an area of 3600 μm^2^. A more detailed inspection of the metasurface, performed by SEM, in Figure 2b revealed that the resulting metasurface showed a fill fraction of 4.5% (calculated considering a region of interest of 90 μm^2^) and an interparticle distance of 1.0 ± 0.4 μm. The PEM was designed to have the PAH as the top-coat layer. The PAH is a cationic PE that promoted the incorporation of AgNCs^27^ via electrostatic attractions involving the stabilizing agent of the AgNCs and the positively charged amine groups of the PAH. Indeed, AgNCs are stabilized by polyvinylpyrrolidone. Thus, they have a negative zeta potential^28^ suitable to be electrostatically incorporated by a cationic PE as the PAH. The PEM not only works to promote the firm incorporation of AgNCs but, remarkably, behaves also as a dielectric spacer, giving rise to a plasmonic gap or plasmonic nanocavity.^14,27^ Plasmonic gaps can be generated by coupling two planar plasmonic entities, separated by a dielectric medium, resulting in a near-perfect absorption at the plasmon resonance arising from the collective electromagnetic response of the plasmonic entities interfering with incident radiation.^14,15^ The spectroscopic analysis of the metasurface reported in Figure 2c, performed using the customized reflective fiber coupled spectrophotometer schematized in Figure 2d (NIR laser switched off), shows the dip in reflectance associated with the AgNCs absorption at 530 nm, evidenced in the yellow stripe (Figure 2c). In addition, a further intense absorption band arises at 764 nm (Figure 2c) due to the metasurface-based absorber (pink stripe). This effect can be explained by assuming that a suitable AgNCs density is achieved, AgNCs interact with the Au layer through the dielectric film (PEM) and behave like magnetic dipoles.

The magnetic dipoles produce a collective action that generates a magnetic response. Consequently, there is an impedance matching (or *n* matching) between the metasurface and the surrounding free space.

As a result, the incident white light radiation exhibits a well-evident absorption band centered at 764 nm because light transmission and reflection are eliminated, and the radiation gets absorbed^14^ (Figure 2c).

**Figure 3:**
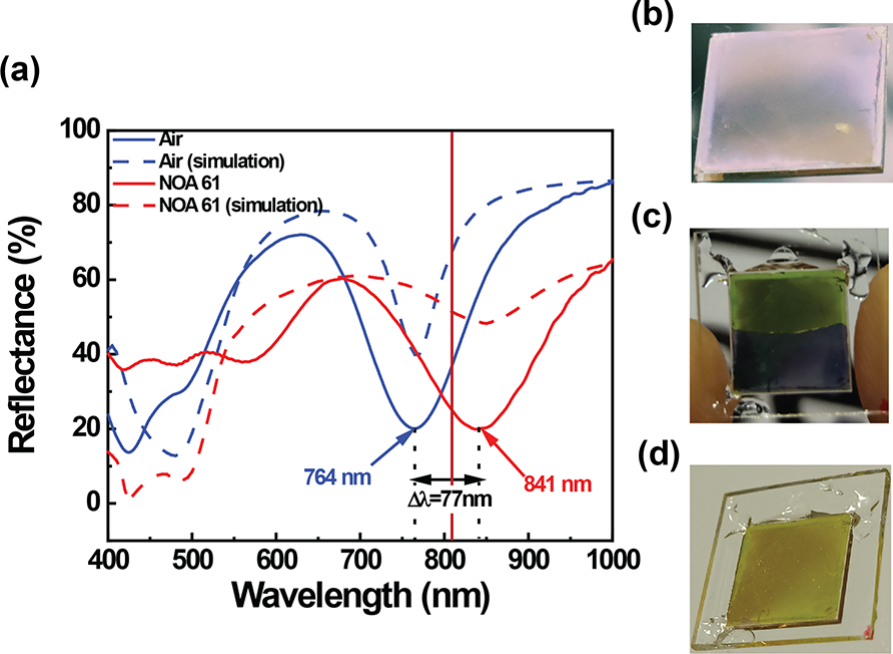
Reflectance spectroscopy characterization (a) of the metasurface cell before (blue, solid curve) and after (red, solid curve) infiltration with NOA 61. The dashed lines of the corresponding color show the results from numerical simulations for the empty cell (dashed blue line) and the cell filled with NOA 61 (dashed red line). The vertical red line indicates the reflectance of the metasurface sample at 808 nm. The optical shift of the metasurface peak accounts for the sensitivity to the *n* change. Photos of the as-prepared metasurface (b) and during (c) and at the end (d) of the infiltration process with NOA 61.

Several experimental parameters interplay to determine the wavelength of the absorption band, including the AgNCs size, the thickness and optical properties of the dielectric spacer, the AgNCs density, and their interparticle distance.^14,15^ The absorption efficiency, defined as the intensity difference between the maximum reflected light (83%) and the reflection at 764 nm, is about 60%. To verify the dependence of the absorption band wavelength on the *n* surrounding the AgNCs, NOA 61, an isotropic material with a high *n* (*n* = 1.56), was utilized. To this end, the metasurface was used to fabricate a glass cell and infiltrated via capillary forces with NOA 61. The glass cell was fabricated by putting the metasurface in contact via 10 μm microbeads with the top cover glass. The presence of the infiltrating medium promoted a red shift of the metasurface absorption band of about 77 nm (from 764 to 841 nm), as reported in Figure 3a (solid blue and red lines).

Remarkably, the resonance shift is also visible as a color change that is perceptible to the naked eye (from the as-prepared metasurface, Figure 3b, to the NOA 61 infiltrated metasurface, Figure 3d). Figure 3c shows the drastic and gradual color alteration induced by the NOA 61 filling the cell by capillarity during the infiltration step. This effect can be understood as follows. The plasmonic modes can be modeled as standing waves living in the cavity formed in the gap between AgNCs and the gold substrate.^16,29,30^ While their resonance wavelengths strongly depend on the geometry of the cavity, gap thickness, and AgNC length as well as the optical properties of the materials forming the cavity, AgNCs, PEM, and substrates, they also depend on the optical properties of the background material. Indeed, open boundaries are generated under the lateral sides of the AgNCs that are in contact with the PEM layer. Here, the plasmonic modes undergo an abrupt alteration of their optical properties due to the change of the dielectric properties from the PEM to the medium surrounding AgNCs, such as air, NOA 61, water, or NLC in this work (see experiments below). Upon reflection, the phase of the plasmonic modes at this interface can be shifted differently according to the background material’s optical properties, affecting the cavity’s resonance wavelength. This effect was confirmed in simulation by reproducing the red shift of the resonance upon infiltration with NOA 61 by simply changing the *n* of the background material from 1 (air) to 1.56 (NOA 61). The dashed lines (blue and red) in Figure 3a show the numerical simulations of the metasurface absorption band before (blue dashed curve) and after (red dashed curve) the NOA 61 infiltration. A control experiment infiltrating the metasurface cell with water (*n* = 1.33) showed a red shift of the metasurface absorption band of 10 nm (Figure SI 1). This shift is smaller than that for NOA 61, which the change of the background *n* cannot fully explain. However, a swelling of the PEM induced by water infiltration would blue-shift the gap plasmonic resonance,^31^ compensating for the red shift induced by the increase of the *n* value of the background material. Indeed, the small 10 nm shift was explained in our simulation by considering a swelling of 2 nm, from 13 nm, of the PEM and a change of the background material *n* (from 1 for air to 1.33 for water).

### Photothermal Investigation

**Figure 4:**
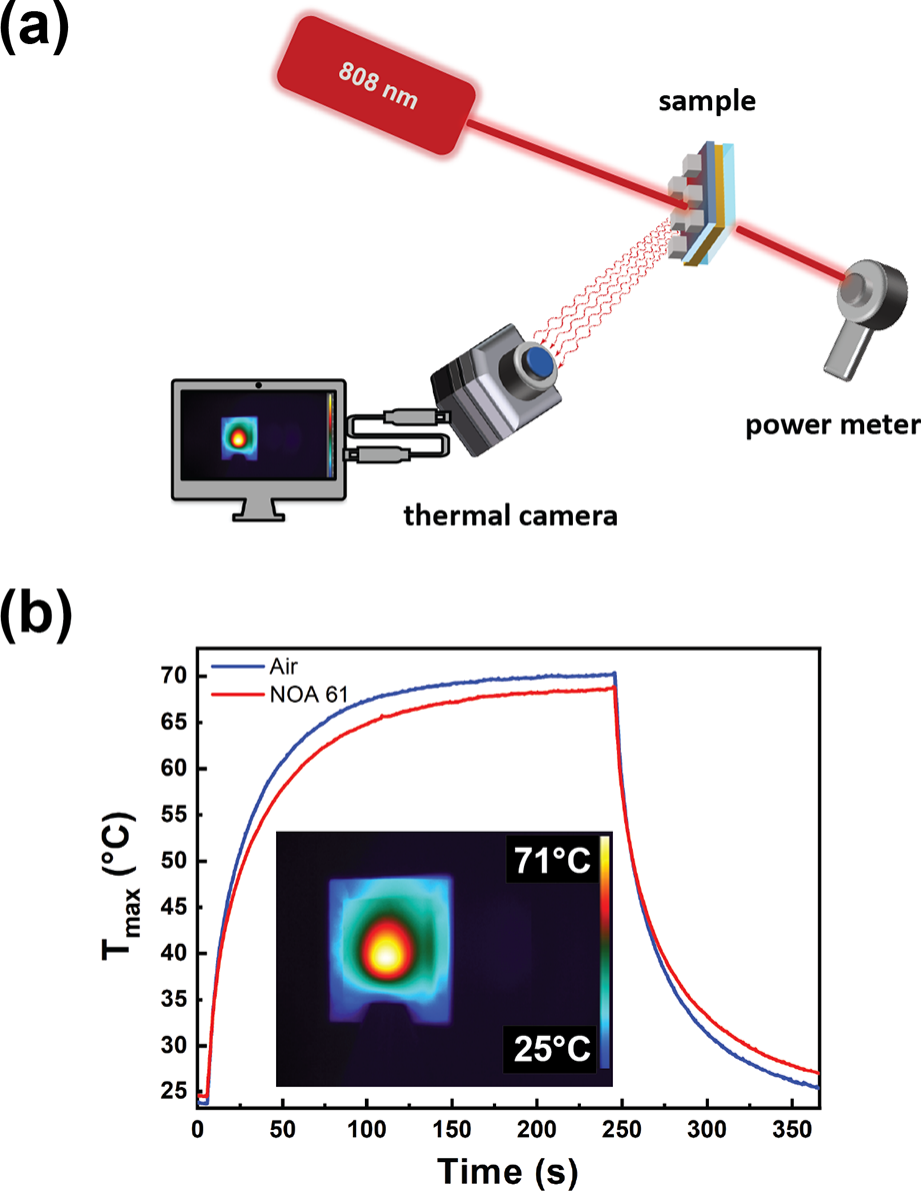
Photothermal investigation of the NOA 61-metasurface sample. Schematic of the thermo-optical setup designed to analyze the photothermal properties of the metasurface: the laser spot is focused on the center of the sample, which is monitored with a thermal camera to record the thermoplasmonic heating upon laser illumination. A power meter is placed behind the metasurface cell to measure the laser intensity (a). Plot reporting the maximum temperature (*T*_max_) values as a function of irradiation time for the metasurface cell infiltrated with air (blue curve) or NOA 61 (red curve) obtained by setting the laser intensity at 8.4 W/cm^2^. The inset reports a thermographic image of the cell infiltrated with NOA 61 acquired before turning off the laser (b).

To achieve a light-controllable active metasurface, it is necessary to investigate the light-to-heat conversion ability of the sample. To this end, the thermo-optical setup, sketched in Figure 4a, was realized. We used it to analyze the selective light absorption of the metasurface at the resonance wavelength. A CW laser beam emitting at 808 nm (NIR laser) with a power density of 8.4 W/cm^2^ was used to illuminate the cell. The time–temperature profile curves, reported in Figure 4b, were obtained from the analysis of thermographic images measured by a high-resolution thermal camera. The inset of Figure 4b shows a representative thermographic image of the NOA 61-metasurface sample taken at the end of the irradiation process, just before switching off the laser. The color scale represents the maximum temperature value (*T*_max_) measured at the center of the sample, corresponding with the laser spot impinging the metasurface. The *T*_max_ values were reported as a function of the irradiation time, giving rise to time–temperature plots reported in Figure 4b. This indicates that the NIR laser irradiation of the empty metasurface cell (blue curve of Figure 4b) produced a progressive temperature increase from 25 to 71 °C during an irradiation period of 240 s.

Such a temperature increase can be associated with light absorption by the metasurface plasmonic band. Indeed, a control experiment demonstrated that by irradiating with the NIR laser (same experimental conditions) a cell fabricated depositing AgNCs on a glass substrate (by following the same procedure used for the metasurface and characterized in Figure SI 2), a temperature variation (Δ*T*_max_) of only 1 °C after 240 s was reached (Figure SI 3, blue curve). Another control experiment (Figure SI 3, red curve) performed using a gold-coated glass substrate that was illuminated with the NIR laser under the same experimental conditions again showed a moderate temperature increase of only 10 °C. When the sample was infiltrated with NOA 61, *T*_max_ reached 69 °C after 240 s of NIR laser irradiation (red curve of Figure 4b). The photothermal efficiency of the metasurface before and after the infiltration with NOA 61 can be estimated based on the time constant value (τ). Such an approach is validated in the Roper model^32^ and is widely accepted for assessing the photothermal efficiency of new materials,^33−36^ as τ is inversely proportional to the photothermal efficiency. The calculated values of τ before and after the infiltration with NOA 61 were 34 and 42 s, respectively (see the Supporting Information for more details). The metasurface infiltration with a high-*n* medium was expected to produce *T*_max_ values higher than those measured for the empty cell, considering the direct dependence of the temperature increase (produced by thermoplasmonic phenomena) from the *n* values.^37^

It is worth mentioning that under the investigated experimental conditions the effect of the *n* increase was counterbalanced by the rise of the reflectance value (lower absorption) at 808 nm. However, as reported in Figure 3a, the reflectance rate at 808 nm decreased from 39% (for the empty cell) to 21% after infiltration with NOA 61.

The reduction in the reflectance (higher absorption) occurs because the utilized optical setup (Figure 2d) collects only the specular reflection. In the actual case, because of the *n* increase, the reflected diffusive light plays a crucial role; therefore, there is an apparent increase in light absorption because the diffusive component is not collected. Therefore, the competition between the *n* increase and the effective reflectance percentage increase produced an overall effect that generates only a 3 °C decrease of the *T*_max_ values upon metasurface cell infiltration with the NOA 61.

### Integration of an NLC Layer for Thermoplasmonic Control of the Optical Properties

The light-to-heat conversion capability of the metasurface (thermo-plasmonic effect) was exploited here to achieve an all-optical control of the metasurface plasmonic band. To this end, the metasurface sample was overlaid with a thermotropic NLC as a suitable medium for actively controlling the plasmonic band.

In particular, the E7-NLC was selected because the nematic to isotropic phase transition temperature (61 °C)^38^ lies in the range of the *T*_max_ values measured during the photothermal characterization of the metasurface.

**Figure 5:**
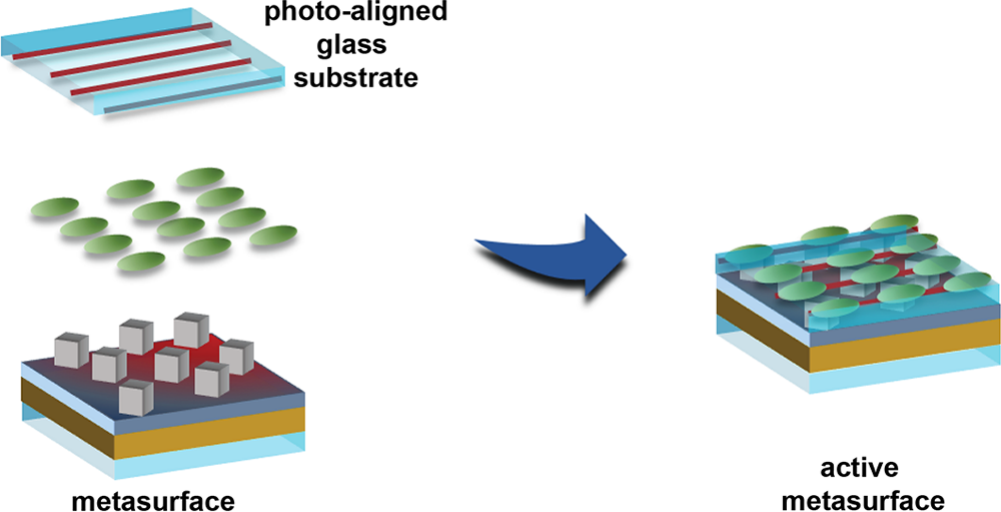
Schematic of the NLC metasurface cell preparation. The NLC is sandwiched between the metasurface and a planarly photoaligned top cover glass substrate.

**Figure 6:**
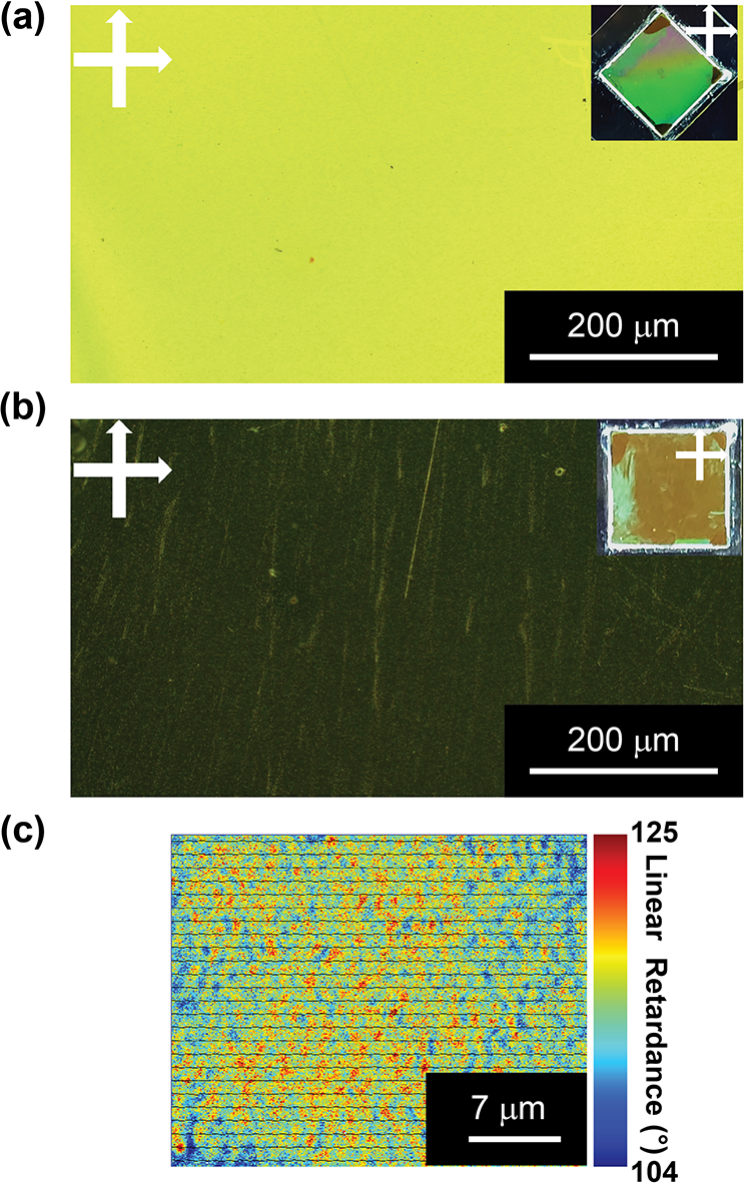
POM view of the NLC metasurface sample between crossed polarizers and the corresponding sample photos (a, b). The molecular director was aligned at 45° (a) and 0° (b) with respect to the polarizer/analyzer axis. Mueller matrix polarimeter characterization of the NLC metasurface sample (c).

Therefore, thermoplasmonic heating can be used to control the phase transition of the NLC film, producing a photothermal control of the metasurface plasmonic band. To induce NLC alignment, PAAD-72 was selected as the photoalignment material. The step-by-step NLC metasurface sample fabrication is sketched in Figure 5, while all the details are reported in the Experimental Section. A uniform planar alignment of the NLC molecules was achieved, as confirmed by the corresponding POM image reported in Figure 6a.

In particular, when the molecular director is aligned at 45° with respect to the polarizer/analyzer axis, the NLC metasurface sample appeared uniform, clearly indicating the uniform alignment of the NLC molecules (Figure 6a). By rotating the sample with the molecular director aligned at 0° (Figure 6b), a dark area is noticed, thus confirming the planar alignment of the NLC film. Interestingly, under these conditions (Figure 6b), a POM micrograph of the sample shows bright spots whose dimensions are compatible with the presence of AgNCs. They can be associated with small NLC domains seeded by the AgNCs. We confirmed this observation by analyzing the sample using a Muller matrix polarimeter (Figure 6c).

**Figure 7:**
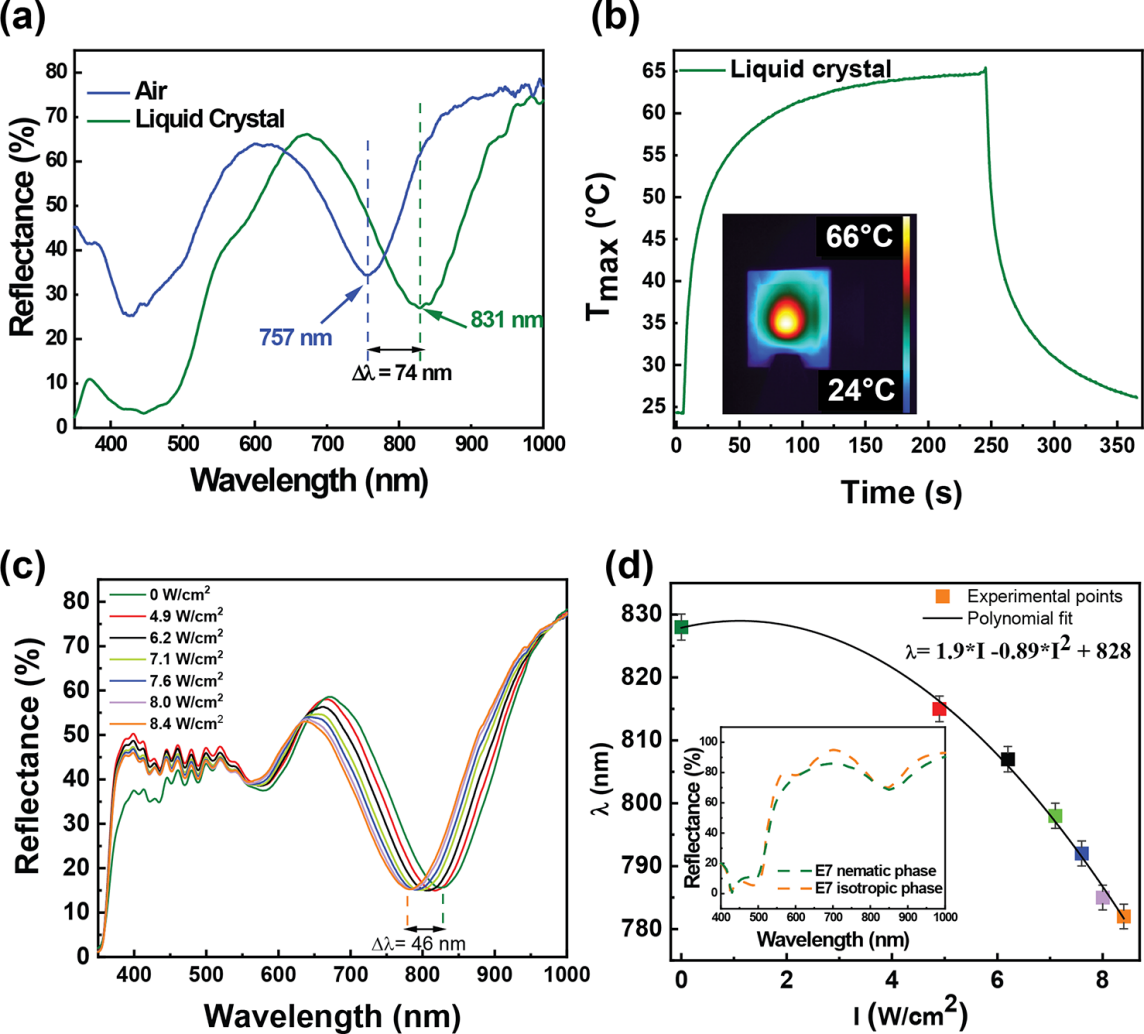
Reflectance spectrum of the NLC metasurface cell (green line) showing a 74 nm red shift with respect to the reflectance spectrum of an empty metasurface cell (blue line) (a). Time–temperature profile of the NLC metasurface cell irradiated with a NIR laser (b). The inset shows a thermographic image of the NLC metasurface, acquired before shutting down the laser. Reflectance spectra of the NLC metasurface cell were measured at different NIR laser power densities. The spectra show the possibility of actively modulating the position of the NLC metasurface absorption band as a function of light intensity. The LC phase can be controlled from a nematic (green line, laser switched off) to an isotropic state (orange line, laser intensity at 8.4 W/cm^2^) induced by the photothermal heating of the metasurface (c). Absorption peak positions are a function of the laser intensity. A calibration curve is extrapolated by fitting the experimental points with a parabolic function. The inset reports numerical simulations of the reflectance spectra for the NLC metasurface cell in the nematic (green dashed line) and isotropic (orange dashed line) states (d).

The retardance is generally uniform, but we associated many red spots (Figure 6c) with partially misaligned NLC areas close to the AgNCs. The unpolarized spectroscopic analysis of the NLC metasurface sample (Figure 7a) highlighted a significative red shift of the metasurface plasmonic band due to the increase of the *n* value of the infiltrating medium from 1 (air) to 1.6 (E7, average *n*).^24^

The wavelength of the metasurface plasmonic band undergoes a red shift from 756 nm (bare metasurface, Figure 7a, blue curve) to 828 nm (NLC metasurface, Figure 7a, green curve). Furthermore, the NLC metasurface sample, under NIR laser irradiation (see the thermo-optical setup in Figure 4a), produced a temperature increase from 24 to 66 °C, resulting in τ values of 34 and 55 s, respectively (see the Supporting Information for more details), before and after the infiltration with the NLC film. The time–temperature profile of the NLC metasurface is reported in Figure 7b. Accordingly, the 240 s of NIR laser exposure was expected to be sufficient to induce the nematic to isotropic transition of the NLC.^38^

To determine the spectral response of the NLC metasurface under laser illumination, we probed the sample area using a customized reflective fiber-coupled spectrophotometer (Figure 2d) while increasing the NIR laser intensity (Figure 7c). In particular, the area investigated by the fiber-coupled spectrophotometer exactly overlapped the photoactivated area of the NLC metasurface so that it was possible to collect the reflectance spectra of the photothermal activated sample area.

Figure 7c points out that by increasing the NIR laser intensity from 0 to 8.4 W/cm^2^, a gradual blue shift of the plasmon band of the NLC metasurface from 828 nm (green trace, laser off) to 782 nm (orange trace, laser intensity 8.4 W/cm^2^) is obtained. This blue shift can be ascribed to a photothermal-induced NLC phase transition that reduces the average *n* of the NLC film (from 1.6 to 1.55). A control experiment reported in Figure SI 4 highlighted that when the metasurface cell was infiltrated with NOA 61 (instead of the NLC), the NIR laser irradiation (8.4 W/cm^2^) did not shift the wavelength of the absorption band, thus confirming that results shown in Figure 7c are due to the photothermal-induced phase transition of the NLC layer. Remarkably, as evidenced in Figure 7c, by modulating the laser intensity from 4.9 to 8.4 W/cm^2^, the 240 s laser exposure gradually blue-shifted the NLC metasurface absorption band wavelength.

Such a result is better highlighted in Figure 7d, where the metasurface absorption band values’ wavelengths are reported as a function of NIR laser intensity. The experimental points were fitted with a parabolic function that allowed us to extrapolate the calibration curve shown in Figure 7d.

Transmission dynamic experiments were performed to investigate and validate the reversibility and the reproducibility of the spectral properties of the thermoplasmonic controlled NLC metasurface. To this end, we used a pump–probe optical setup (Figure SI 5) equipped with a light source emitting at 650 nm (Figure SI 6a) as a probe. As a result (Figure SI 6b), the transmitted intensity of the NLC metasurface can be reversibly switched from a low-intensity value to a high-intensity value (pump beam on, 8.4 W/cm^2^) and vice versa (pump beam off), as expected from typical intensity oscillations associated with the photoinduced phase transition of a planarly aligned NLC film. The result reported in Figure SI 6b highlights the reversibility and reproducibility of the NLC metasurface sample’s spectral properties, as also confirmed by the photothermal cycling experiments in Figure SI 7. The NLC metasurface absorption position repeatability was further investigated by reporting the NLC metasurface peak position wavelengths as a function of the NIR laser intensity (Figure SI 8). The two sets of measurements, performed by increasing and decreasing the pump laser intensity, respectively, highlighted that the resulting curves overlapped closely, exhibiting a small (a few percentage) hysteresis area due to the nonlinear properties of the NLC layer.

This set of experimental results demonstrated the possibility of achieving a dynamically tunable metasurface by a lithography-free method. Indeed, due to the photothermal properties of the metasurface and the integration with the NLC, it was possible to modulate the metasurface absorption band position in a wavelength range of 46 nm by simply varying the NIR laser intensity.

The corresponding numerical simulations, reported in the inset of Figure 7d, confirm the same trend (blue shift) of the plasmon band of the NLC metasurface that undergoes a phase transition of about 10 nm (from 850 nm, green curve, to 840 nm, orange curve). The difference between experiments and theory can be explained by considering that the theoretical model did not include several aspects, such as heat capacitance anisotropy, light scattering, *n* variation of the PEM, etc.

Remarkably, as detailed in Table SI 1, it is worth noting that the 46 nm dynamic range of the proposed NLC metasurface is comparable to the range resulting from several other triggering mechanisms, such as electrical- or temperature-driven mechanisms achieved by other metasurfaces obtained by conventional nanofabrication methods.

Future efforts will be devoted to modifying the theoretical model to improve the agreement between theory and experiments and to inducing the dynamic control of metasurface optical properties using more affordable sources such as white light lamps.^39^

## Conclusions

We have reported on the fabrication and characterization of a light-controlled optical absorber that exploits the combination of a lithography-free colloidal metasurface and an NLC film. AgNCs, self-assembled and immobilized on a 50 nm thick gold layer using PEM as a dielectric spacer, produce a metasurface-based optical component. The resulting optical absorber shows a well-defined absorption band centered in the NIR range of the electromagnetic spectrum, high absorption efficiency (∼60%), and excellent photothermal properties. The latter has been used to control the *n* value of an NLC layer, thus providing tunability (46 nm), reversibility, and reproducibility to the spectral properties of the realized metasurface-based optical absorber.

Detailed thermographic studies and spectroscopic investigations highlight the extraordinary capability of the active metasurface-based optical absorber to be utilized as a controllable light attenuator. Numerical simulations performed using COMSOL validate the experimental results in terms of the spectral position of the absorption band and *n* sensitivity. Ongoing and future studies are devoted to improving the correlation between simulations and experiments by considering other parameters, such as heat capacitance anisotropy and light scattering. In addition, NLCs with higher birefringence (*n* ≈ 0.5–0.7) will be used for increasing the spectral shift of the metasurface plasmonic band under resonant laser illumination. Our findings open the way for realizing a new class of light-controllable optical components that can be easily used for applications such as optical communications and light harvesting.

## Supporting Information Available

Reflectance spectroscopy characterization of the metasurface cell before and after the infiltration with water; SEM characterization of an indium tin oxide substrate functionalized with AgNCs by following the same metasurface preparation procedure, time–temperature profile of the gold layer and AgNCs array, respectively, irradiated with the NIR laser; reflectance spectra of the metasurface cell infiltrated with NOA 61 and measured without and with NIR laser irradiation; calculation of time constant values; scheme of the optical setup for the transmission dynamic experiments; spectral properties of the 650 nm light source used for transmission dynamic experiments; transmission dynamic experiments; time–temperature profile plots by switching on and off the NIR laser source; absorption peak positions measured by increasing (black squares) and decreasing (red dots) the laser intensity; comparison table that reports the Δλ variation for different NLC-based metasurfaces (PDF)
