# Supplementary material for: Thermoplasmonic Controlled Optical Absorber Based on a Liquid Crystal Metasurface
Source: ACS Appl Mater Interfaces. 2023 Oct 10;15(42):49468–77. doi: 10.1021/acsami.3c09896 (PMC10614192; doi:10.1021/acsami.3c09896)
Supplement: Supplementary file 1 — am3c09896_si_001.pdf [file am3c09896_si_001.pdf]

Supporting Information

Thermo-plasmonic controlled optical absorber based on a liquid crystal metasurface

Francesca Petronella[†], Tristan Madeleine[‡], Vincenzo De Mei[§], Federica Zaccagnini[§], Marinella Striccoli[¥], Giampaolo D'Alessandro[‡], Mariacristina Rumi[&], Jonathan Slagle[&], Malgosia Kaczmarek[#], and Luciano De Sio[§,$]**

[†]National Research Council of Italy, Institute of Crystallography, CNR-IC, Rome Division, Area della Ricerca Roma 1 Strada Provinciale 35d, n. 9 - 00010 Montelibretti (RM)

[‡]School of Mathematical Science, University of Southampton, Southampton SO17 1BJ, United Kingdom

[§]Department of Medico-Surgical Sciences and Biotechnologies Sapienza University of Rome, Latina, Italy

[¥]National Research Council of Italy, Institute of Chemical and Physical Processes, CNR-IPCF Bari Division, Via Orabona 4, 70126 Bari, Italy

[&]Materials and Manufacturing Directorate, Air Force Research Laboratory, Wright-Patterson Air Force Base, Ohio 45433-7707, USA

[#]School of Physics and Astronomy, University of Southampton, Southampton SO17 1BJ, United Kingdom

[$]National Research Council of Italy, Licryl, Institute NANOTEC, Arcavacata di Rende, Italy

Corresponding authors: mfk@soton.ac.uk; luciano.desio@uniroma1.it

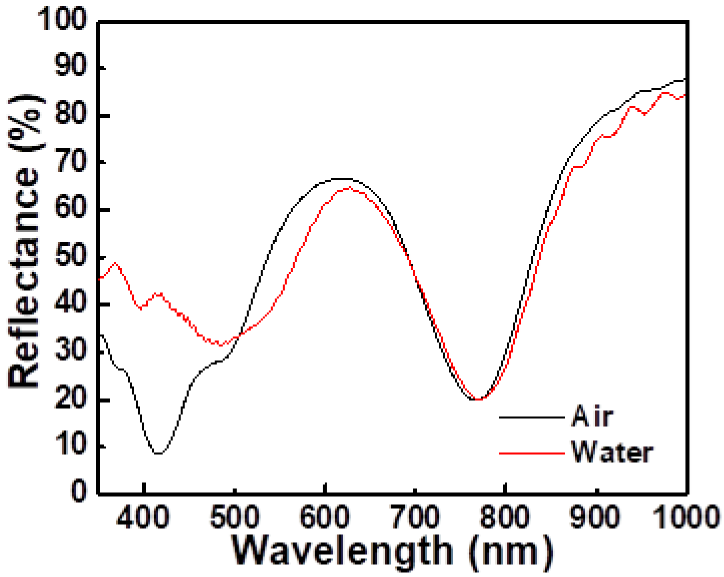

Figure SI 1. Reflectance spectroscopy characterization of the metasurface cell before (black trace) and after (red trace) the infiltration with water.

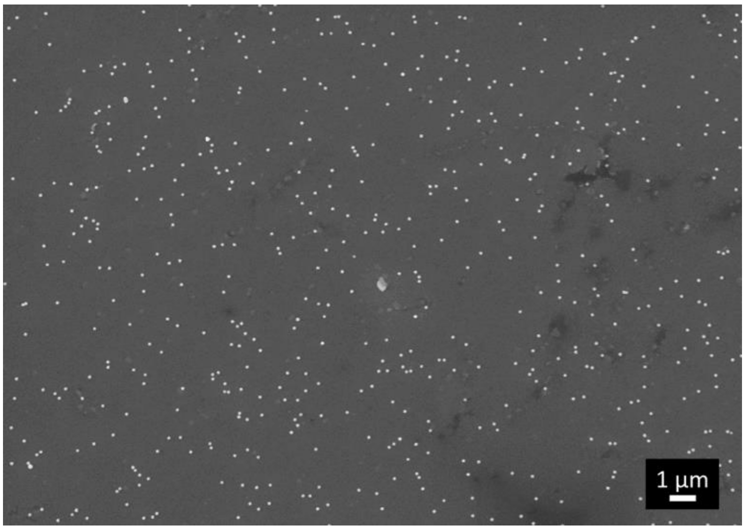

Figure SI 2. SEM characterization of an indium tin oxide (ITO) substrate, functionalized with AgNCs by carrying out the same procedure followed for the metasurface preparation. The SEM micrograph was performed with a field emission SEM, FE-SEM Zeiss-Sigma, Carl Zeiss Co., Oberkochen, Germany, operating at 10 KV, working distance 3.9 mm.

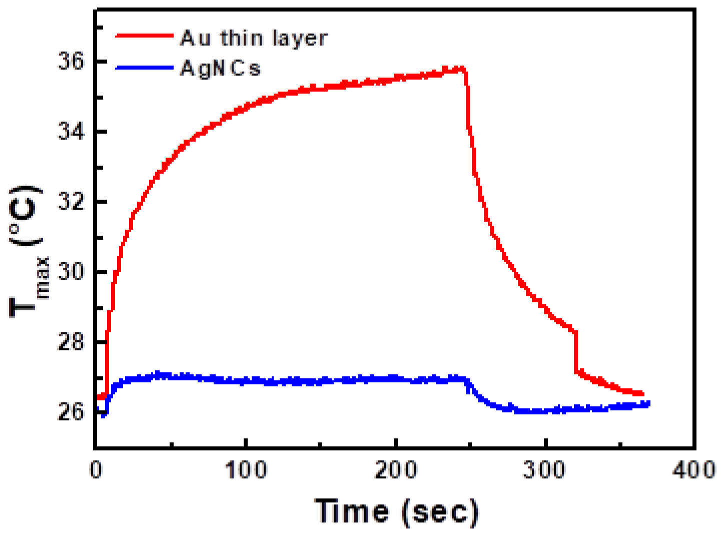

Figure SI 3. Time-temperature profile of the gold layer (red curve) and AgNCs (blue curve) array irradiated with the NIR laser at 8.4 W/cm^2.

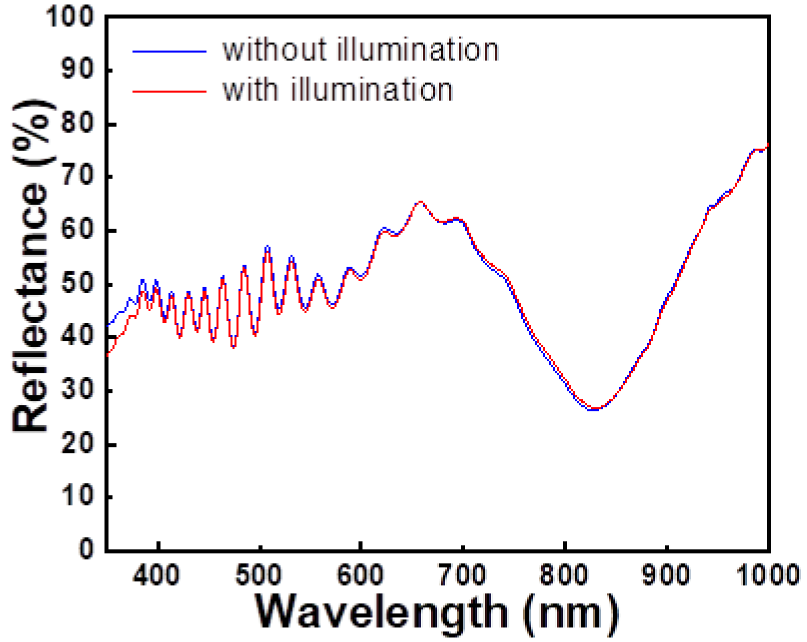

Figure SI 4. Reflectance spectra of the metasurface cell infiltrated with NOA 61 and measured without (blue trace) and with (red trace) NIR laser irradiation at an intensity of 8.4 W/cm^2 for 240s.

Calculation of the time constant values

To evaluate the photo-thermal conversion efficiency (η), the time constant values τ were used. Such a constant is related to the η values, in agreement with the Roper model (equation 1).[1] The time constant values τ are often accepted in the literature to estimate the photo-thermal conversion efficiency of nanomaterials.[2-4]

Indeed, η is defined as:

$$\eta = \frac{hS(T_{max} - T_{sur}) - Q_0}{I(1 - 10^{-A_{808}})} \qquad \text{equation 1}$$

while hS (the heat transfer coefficient) is defined as:

$$hS = \frac{m_s - c_s}{\tau} \qquad \text{equation 2}$$

where m_s and c_s are the mass and heat capacity of the solution while τ is the time constant. Therefore, according to equations 1 and 2, the η is inversely proportional to the τ.

The τ values were calculated as the reciprocal of the slope of the linear fit, obtained by plotting the -lnθ as a function of irradiation time (t in equation 3):

$$-ln\theta = \frac{t}{\tau} \qquad \textit{equation 3}$$

where θ is defined as:

$$\theta = \frac{T(t) - T_{sur}}{T_{max} - T_{sur}} \qquad \textit{equation } 4$$

In equation 4 T(t) is the temperature registered during the cooling phase at the time t,

T_{sur} is the room temperature at the sample surface, and T_{max} is the maximum temperature.

The values of τ were calculated for the metasurface before and after the infiltration with NOA 61. It turns out that the τ values are 34 s and 42 s with and without NOA 61, respectively. Furthermore, the value of τ was also calculated for the NLC metasurface, and the result was 55 s.

Transmission dynamic experiments

The reversibility and the reproducibility of the spectral properties of the NLC metasurface were investigated by performing transmission dynamic experiments. To this end, the optical setup in Figure SI 5 was realized. The setup is designed so that the sample is probed by using a 650 nm light source obtained by placing a 650 nm pass band color filter in front of a white light source while turning on and off the pump NIR laser source at the highest intensity (I=8.4 W/cm^2).

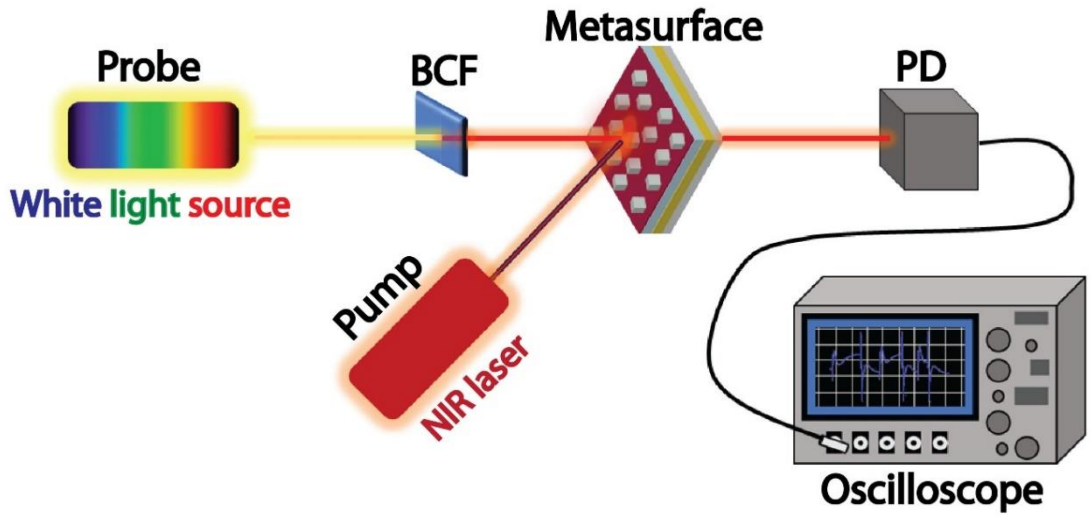

Figure SI 5. Schematic of the optical setup for the transmission dynamic experiments. A 650 nm pass band color filter (BCF) was placed in front of the white light source to probe the metasurface while turning on and off the pump NIR laser with intensity I=8.4 W/cm^2. The transmitted intensity was measured by the photo-detector (PD) and collected by the oscilloscope.

The 650 nm light source (the spectral features are shown in Figure SI 6a) was selected because in this spectral range, the NLC metasurface sample (Figure 7c) shows an intensity variation while increasing the pump beam intensity value.
In addition, at this wavelength value, the sample exhibits a transmitted intensity value that can be measured by the mean of a conventional photo-detector, making the optical setup (Figure SI 5), very simple to realize.

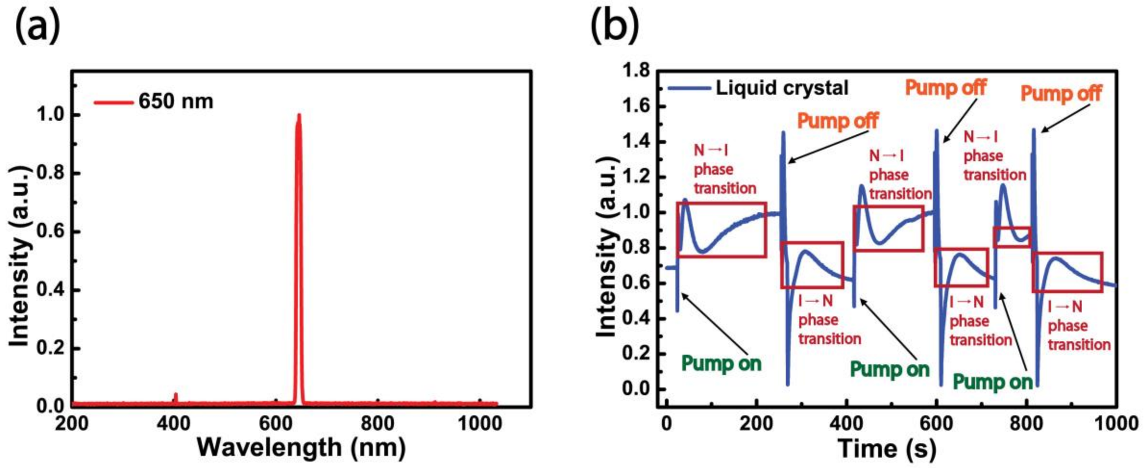

Figure SI 6. (a) Spectral properties of the 650 nm light source used to perform the transmission dynamic experiments(a). Dynamic of the transmitted intensity while switching ON and OFF the pump NIR laser for 3 cycles. (b) The NLC phase transitions from nematic to isotropic state and vice versa are highlighted with a red box.

It turns out (Figure SI 6b) that the transmitted intensity can be reversibly triggered from a low-intensity value to a high-intensity value (pump beam on) and vice versa (pump beam off), evidencing in between the typical

intensity oscillations associated with the photo-induced phase transition of an NLC film planarly aligned. The intensity increase upon the illumination of the pump beam is consistent with the reflectivity reduction at 650 nm, as seen in Figure 7c. The result reported in Figure SI 6b highlights the reversibility and the reproducibility of the spectral properties of the NLC metasurface sample.

To further highlight the NLC metasurface sample's reversibility and reproducibility, we have performed photo-thermal cycling experiments by monitoring the time-temperature evolution while turning on and off the NIR pump beam. As shown in Figure SI 7, for three consecutive cycles, the temperature increase was fully reproducible without any sign of irreversibility. This further characterization, once again, confirms the reversibility of the realized NLC metasurface sample.

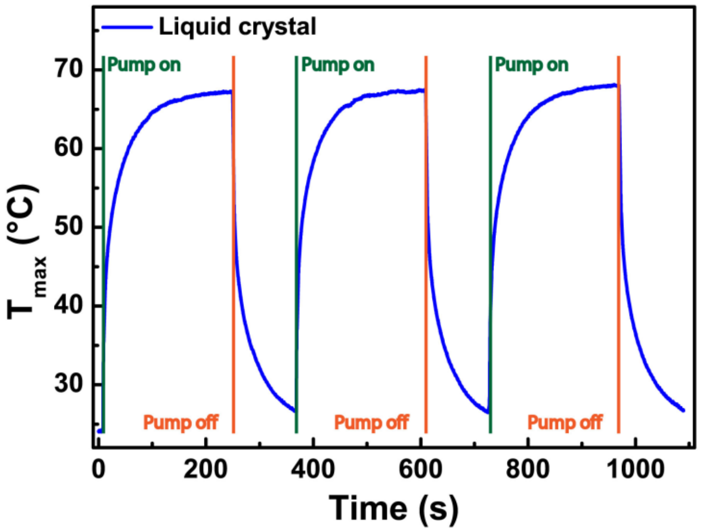

Figure SI 7. Time-temperature profile by switching on and off the NIR laser source for 3 cycles. Each cycle includes 5 seconds of initial state, 4 minutes of NIR irradiation, and 2 minutes of cooling.

Absorption peak position repeatability

To assess the repeatability of the absorption peak position of the NLC metasurface as a function of NIR laser irradiation intensity, we performed a specific experiment consisting of two consecutive steps. In the first, we measured the NLC metasurface reflectance spectra by increasing the laser intensity values, and in the second by decreasing the laser intensity values. The results are summarized in Figure SI 8 and reported as peak wavelength values as a function of the NIR laser intensity for the first step (black dots, increasing intensity values) and for the second step (red squares, decreasing intensity values), resulting in one entire cycle.

As pointed out in Figure SI 8, the values obtained for the two consecutive steps of the cycle almost overlapped, exhibiting a small (a few percentage) hysteresis area because of the nonlinear properties of the NLC layer.

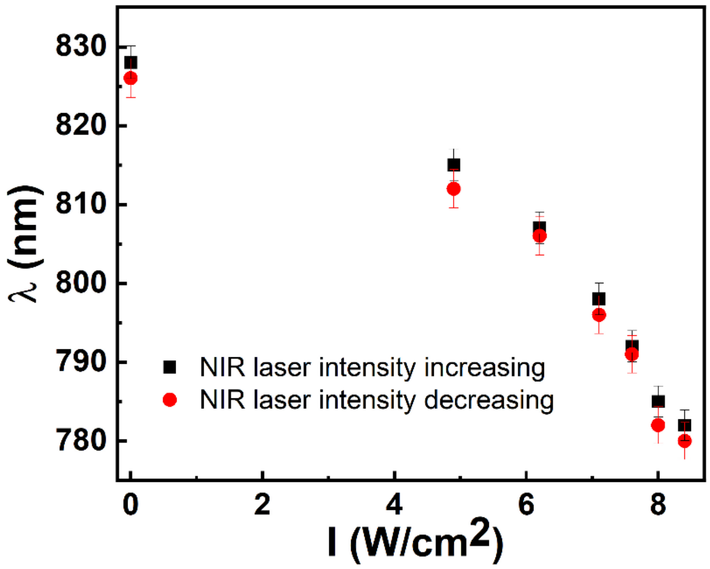

Figure SI 8. Absorption peak positions by increasing (black squares) and decreasing (red dots) the laser intensity.

Comparison table of the dynamic range values

Table S1. Comparison table that reports the Δλ variation for different NLC-based metasurfaces according to the specific mechanism utilized to activate the sample.

Mechanism	Reference	Δλ
Electrically tunable metasurface	6	101 nm<br>(from 0 V to 5 V)
Electrically tunable metasurface	7	65 nm<br>(from 0 V to 10 V)
Electrically tunable metasurface	8	≈ 110 nm<br>(from 0 V to 2.7 V)
Electrically tunable metasurface	9	49 nm<br>(from 0 V to 10 V)
Temperature tunable metasurface	10	40 nm<br>(from 21 °C to 61 °C)
Electrically tunable metasurface	11	50 nm and 25 nm<br>(from 0 V to 70 V)
Temperature tunable metasurface	12	10 nm<br>(from 20 °C to 80 °C)

References

(1) Roper, D. K.; Ahn, W.; Hoepfner, M. Microscale Heat Transfer Transduced by Surface Plasmon Resonant Gold Nanoparticles. *J. Phys. Chem. C Nanomater. Interfaces* **2007**, *111* (9), 3636–3641.

(2) Zhang, K.; Ma, Z.; Li, S.; Zhang, W.; Foda, M. F.; Zhao, Y.; Han, H. Platelet-Covered Nanocarriers for Targeted Delivery of Hirudin to Eliminate Thrombotic Complication in Tumor Therapy. *ACS Nano* **2022**, *16* (11), 18483–18496.

(3) Xu, Y.; Li, C.; Wu, X.; Li, M.-X.; Ma, Y.; Yang, H.; Zeng, Q.; Sessler, J. L.; Wang, Z.-X. Sheet-like 2D Manganese(IV) Complex with High Photothermal Conversion Efficiency. *J. Am. Chem. Soc.* **2022**, *144* (41), 18834–18843.

(4) Vischio, F.; Carrieri, L.; Bianco, G. V.; Petronella, F.; Depalo, N.; Fanizza, E.; Scavo, M. P.; De Sio, L.; Calogero, A.; Striccoli, M.; Agostiano, A.; Giannelli, G.; Curri, M. L.; Ingrosso, C. Au Nanoparticles Decorated Nanographene Oxide-Based Platform: Synthesis, Functionalization and Assessment of Photothermal Activity. *Biomater. Adv.* **2023**, *145* (213272), 213272.

(5) Fanizza, E.; Mastrogiacomo, R.; Pugliese, O.; Guglielmelli, A.; De Sio, L.; Castaldo, R.; Scavo, M. P.; Giancaspro, M.; Rizzi, F.; Gentile, G.; Vischio, F.; Carrieri, L.; De Pasquale, I.; Mandriota, G.; Petronella, F.; Ingrosso, C.; Lavorgna, M.; Comparelli, R.; Striccoli, M.; Curri, M. L.; Depalo, N. NIR-Absorbing Mesoporous Silica-Coated Copper Sulphide Nanostructures for Light-to-Thermal Energy Conversion. *Nanomaterials (Basel)* **2022**, *12* (15), 2545.

(6) Sharma, M.; Hendler, N.; Ellenbogen, T. Electrically Switchable Color Tags Based on Active Liquid-crystal Plasmonic Metasurface Platform. *Adv. Opt. Mater.* **2020**, *8* (7), 1901182.

(7) Sharma, M.; Michaeli, L.; Haim, D. B.; Ellenbogen, T. Liquid Crystal Switchable Surface Lattice Resonances in Plasmonic Metasurfaces. *ACS Photonics* **2022**, 9 (8), 2702–2712.

(8) Buchnev, O.; Podoliak, N.; Kaczmarek, M.; Zheludev, N. I.; Fedotov, V. A. Electrically Controlled Nanostructured Metasurface Loaded with Liquid Crystal: Toward Multifunctional Photonic Switch. *Adv. Opt. Mater.* **2015**, *3* (5), 674–679.

(9) Chang, X.; Pivnenko, M.; Shrestha, P.; Wu, W.; Zhang, W.; Chu, D. Electrically Tuned Active Metasurface towards Metasurface-Integrated Liquid Crystal on Silicon (Meta-LCoS) Devices. *Opt. Express* **2023**, *31* (4), 5378.

(10) Sautter, J.; Staude, I.; Decker, M.; Rusak, E.; Neshev, D. N.; Brener, I.; Kivshar, Y. S. Active Tuning of All-Dielectric Metasurfaces. *ACS Nano* **2015**, *9* (4), 4308–4315.

(11) Komar, A.; Fang, Z.; Bohn, J.; Sautter, J.; Decker, M.; Miroshnichenko, A.; Pertsch, T.; Brener, I.; Kivshar, Y. S.; Staude, I.; Neshev, D. N. Electrically Tunable All-Dielectric Optical Metasurfaces Based on Liquid Crystals. *Appl. Phys. Lett.* **2017**, *110* (7), 071109.

(12) Parry, M.; Komar, A.; Hopkins, B.; Campione, S.; Liu, S.; Miroshnichenko, A. E.; Nogan, J.; Sinclair, M. B.; Brener, I.; Neshev, D. N. Active Tuning of High-Q Dielectric Metasurfaces. *Appl. Phys. Lett.* **2017**, *111* (5), 053102.
